# A new genus of Ptiloneuridae, its position within the family, and descriptions of five species (Psocodea, ‘Psocoptera’)

**DOI:** 10.3897/zookeys.780.26753

**Published:** 2018-08-08

**Authors:** Ranulfo González-Obando, Alfonso N. García Aldrete, Nancy Carrejo-Gironza, Julian Mendivil

**Affiliations:** 1 Grupo de Investigaciones Entomológicas (GIE), Departamento de Biología, Facultad de Ciencias Naturales y Exactas, Universidad del Valle, Santiago de Cali, Colombia Universidad del Valle Santiago de Cali Colombia; 2 Departamento de Zoología, Instituto de Biología, Universidad Nacional Autónoma de México, Apdo. Postal 70-153, CdMx, México Universidad Nacional Autónoma de México Mexico Mexico

**Keywords:** biodiversity, Colombia, neotropics, taxonomy

## Abstract

Upon examination of ptiloneurid specimens recently collected in forests of natural areas and Natural National Parks in Colombia, ten males and seven females were found that represent five species of an undescribed ptiloneurid genus. It differs from the other genera in the family by characters of the wings, hypandrium, phallosome, epiproct, female gonapophyses, and sternum IX. An identification key to the males of the genera of Ptiloneuridae, and a key to the species of the new genus are presented. A test on the validity and monophyly of the new genus, and its position within the family was also conducted.

## Introduction

Ptiloneuridae, a family of Psocodea (Psocomorpha: Epipsocetae), presently includes the genera *Brasineura* Silva-Neto & García Aldrete, *Euplocania* Enderlein, *Loneura* Navás, *Loneuroides* García Aldrete, *Omilneura* García Aldrete, *Perucania* New & Thornton, *Ptiloneura* Enderlein, *Ptiloneuropsis* Roesler, *Timnewia* García Aldrete, *Triplocania* Roesler, and *Willreevesia* García Aldrete.

Within the framework of the project “Revisión Taxonómica y Endemismo de los Psócidos (Insecta: Psocodea: ‘Psocoptera’) de Areas Protegidas de Colombia”, 17 specimens of Ptiloneuridae, corresponding to five species that could not be assigned to any of the known ptiloneurid genera were found. These specimens were collected in localities of Valle del Cauca, Meremberg Nature Reserve and La Candelaria (Huila), Planes de San Rafael Nature Reserve (Risaralda), National Natural Park Tamá (Norte de Santander), and Chicaque Nature Reserve (Cundinamarca) (Figure 48). Here we describe and illustrate these new species, erect a new genus for them, and discuss its position within the family.

## Materials and methods

Seventeen specimens were available for study, ten males and seven females. Nine specimens were dissected in 80% ethanol, and their parts (head, right legs and wings, and genitals), were mounted in Canada balsam, following standard procedures. Before dissecting, whole specimens were placed in 80% ethanol and observed at 50× to record color. Standard measurements were taken with a filar micrometer, and are given in mm. Abbreviations of parts measured are as follows: FW and HW: right fore- and hind- wing lengths, F, T, t1, t2 and t3: lengths of femur, tibia and tarsomeres 1, 2 and 3 of right hind leg, f1-fn: lengths of flagellomeres 1-n, Mx4: length of fourth segment of right maxillary palp, IO: minimum distance between compound eyes, D and d: antero-posterior and transverse diameter, respectively, of right compound eye, all in dorsal view of head. PO: d/D, H: head length, MxW: maximum head width, Ratio head length (H)/(D), L/W: forewing length/forewing width, lp/wp: pterostigma length/pterostigma width, al/ah: areola postica length/tall, l/w: hindwing length/hindwing width. The specimens studied are deposited in the Entomological Museum, Universidad del Valle (**MUSENUV**), Santiago de Cali, Colombia.

To test the validity and the monophyly of the new genus, and to establish its position within the family, a cladistic analysis was performed, using the free Software TNT 1.1 (Goloboff et al. 2008a) under implied weight schemes, between *K* = 1–9, determines how strongly homoplasious characters are downweighted, lower *K* values indicate that homoplastic characters are more strongly penalized or given less weight (Goloboff 1993) and given the concavity constant value *K* should be calculated as a function of N, which is the ratio of a single extra step at the cost of the most homoplastic character (Goloboff et al. 2008b), we obtained *K* values through a TNT script (setk.run) written by Salvador Arias to calculate an appropriate value for *K*. The script returned a value of *K* = 2 for our data set, which was then employed. Given the purpose of the analysis, only one outgroup, *Spurostigma* Eertmoed (Spurostigmatidae) was used (see Yoshizawa 2002; García Aldrete 2005; Casasola González 2006; Silva-Neto et al. 2016). The characters were treated as nonadditive and the topologies were evaluated with an implicit weight (IW) analysis, using Traditional Search with the algorithm TBR (100 repl/10 TperRp), with1000 replicas and Traditional Search with Bootstrap and Symmetric Resampling (Goloboff et al. 2003). The support of the clades was calculated with ACCTRAN and DELTRAN optimization in WINCLADA ver 1.00.08 (Nixon 2002). The resulting strict consensus tree was exported and the names were edited in CorelDraw X7.

As in Silva Neto et al. (2016b), only one species was used to represent each genus, except for *Triplocania* (two species) and *Colocania* gen. n., in which the males (four species) of each species were included in the analysis. The 17 species included were: *Brasineuradiamantina* Silva Neto & García Aldrete, *Colocaniacandelaria* sp. n., *C.chicaque* sp. n., *C.occidentalis* sp. n., *C.pilosa* sp. n., *Euplocaniacerata* New, *Loneurajinotegaensis* García Aldrete, *Loneuroidescolombianus* García Aldrete, González & Carrejo, *Omilneuracircumvittata* García Aldrete, *Perucanialongiareola* New & Thornton, *Ptiloneurabidorsalis* Enderlein, *Ptiloneuropsisdiamantina* Silva Neto, García Aldrete & Rafael, *Spurostigmacaatinga* Silva Neto & García Aldrete, *Timnewiagreeni* (New), *Triplocaniacervantesi* García Aldrete, *Triplocaniamagnifica* Roesler and *Willreevesiadominica* García Aldrete.

**Table 1.** Data matrix of morphological characters used for the cladistic analysis of Ptiloneuridae and outgroup (*S.caatinga*). For characters 1–19 see Silva-Neto et al. (2016).

	**Character**																						
	**1**	**2**	**3**	**4**	**5**	**6**	**7**	**8**	**9**	**10**	**11**	**12**	**13**	**14**	**15**	**16**	**17**	**18**	**19**	**20**	**21**	**22**	**23**
* S. caatinga *	0	0	0	0	0	0	0	0	0	0	0	-	0	0	0	0	0	0	0	0	0	0	0
* B. diamantina *	1	1	2	0	0	0	0	0	0	0	1	0	0	0	0	0	0	0	1	0	1	0	0
* C. candelaria *	0	3	2	0	0	0	0	0	1	0	1	1	0	1	1	1	0	0	0	1	2	1	1
* C. chicaque *	0	3	2	0	0	0	0	0	-	0	1	1	0	1	1	0	0	0	0	1	2	1	1
* C. occidentalis *	0	2	1	0	0	0	0	0	-	0	1	0	0	1	1	1	0	0	0	1	2	1	1
* C. pilosa *	0	2	2	0	0	0	0	0	1	1	1	0	0	1	1	1	0	0	0	1	2	1	1
* E. cerata *	0	0	1	0	0	0	0	0	1	0	1	1	0	1	0	1	0	0	0	0	1	0	0
* L. colombianus *	0	3	2	1	0	0	0	1	2	2	1	0	1	1	1	0	1	1	0	0	2	0	0
* L. jinotegaensis *	0	3	3	0	0	0	0	0	1	2	1	0	1	1	1	0	1	1	0	0	1	0	0
* O. circumvittata *	0	0	3	0	0	0	0	0	1	1	0	0	0	1	1	1	1	0	0	0	1	0	0
* P. bidorsalis *	0	4	5	0	0	0	0	0	2	2	1	0	1	1	1	0	1	1	0	0	1	0	0
* P. diamantina *	0	3	2	0	0	0	1	0	1	0	1	0	0	0	1	0	1	1	0	0	1	1	0
* P. longiareola *	0	0	0	0	0	0	0	0	3	3	0	0	0	1	1	0	1	0	0	0	0	0	0
* T. cervantesi *	0	0	0	0	0	0	0	0	1	1	0	0	0	0	0	0	1	0	0	0	1	0	0
* T. magnifica *	0	0	0	0	0	0	0	0	1	1	0	2	0	1	0	0	0	0	0	0	1	0	0
* T. greeni *	0	0	1	1	1	1	0	1	2	2	0	1	0	1	0	0	1	0	0	0	0	0	0
* W. dominica *	0	0	2	0	0	0	0	0	1	4	1	0	0	0	1	1	0	1	0	0	1	0	0

**Figures 1–6.**
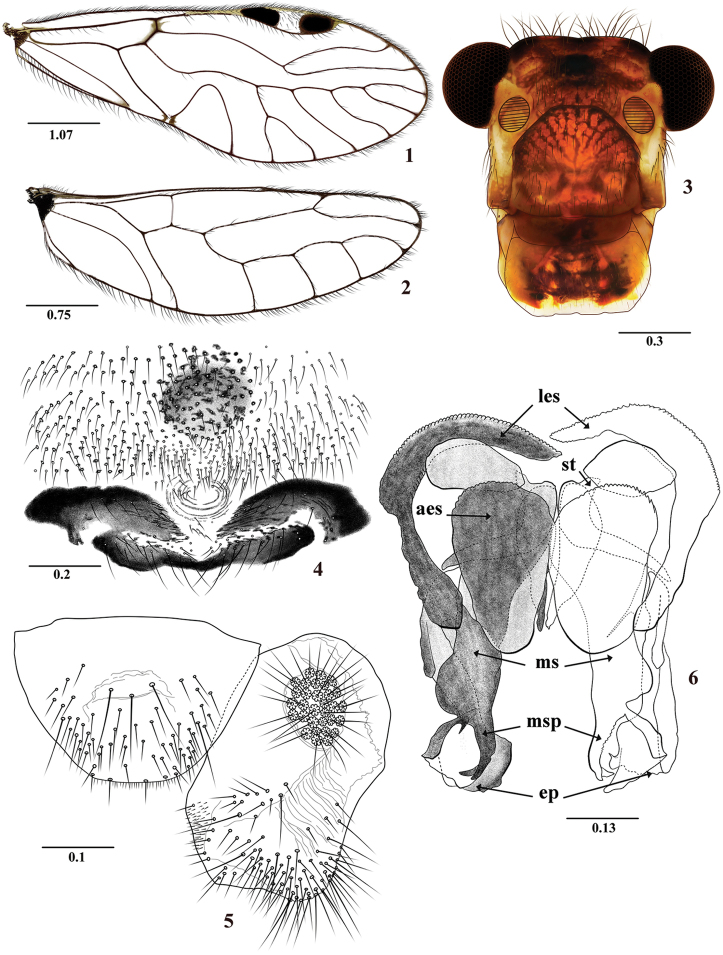
*Colocaniacandelaria* sp. n. Male. **1** Forewing **2** Hindwing **3** Front view of head **4** Hypandrium **5** Left paraproct and epiproct **6** Phallosome. Abbreviations: (aes) anterior endophallic sclerites, (ep) external parameres, (les), lateral endophallic sclerite, (ms) mesal sclerite, (msp) mesal sclerite processes, (st) side struts. Scale bars in mm.

**Figures 7–12.**
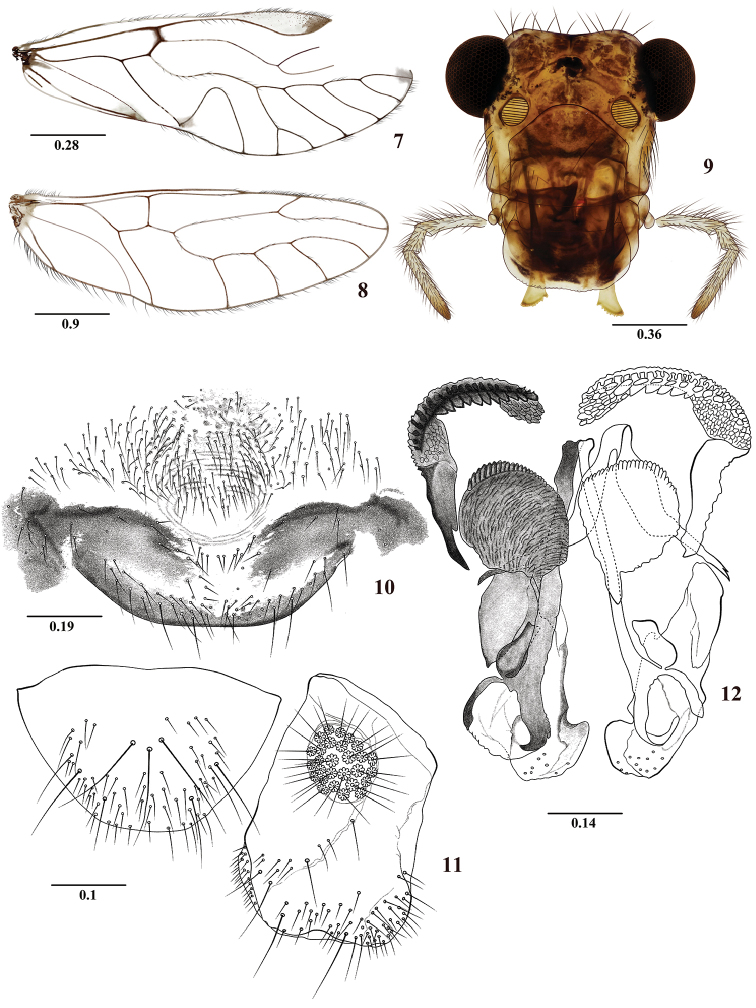
*Colocaniachicaque* sp. n. Male. **7** Forewing **8** Hindwing **9** Front view of head **10** Hypandrium **11** Left paraproct and epiproct **12** Phallosome. Scale bars in mm.

**Figures 13–18.**
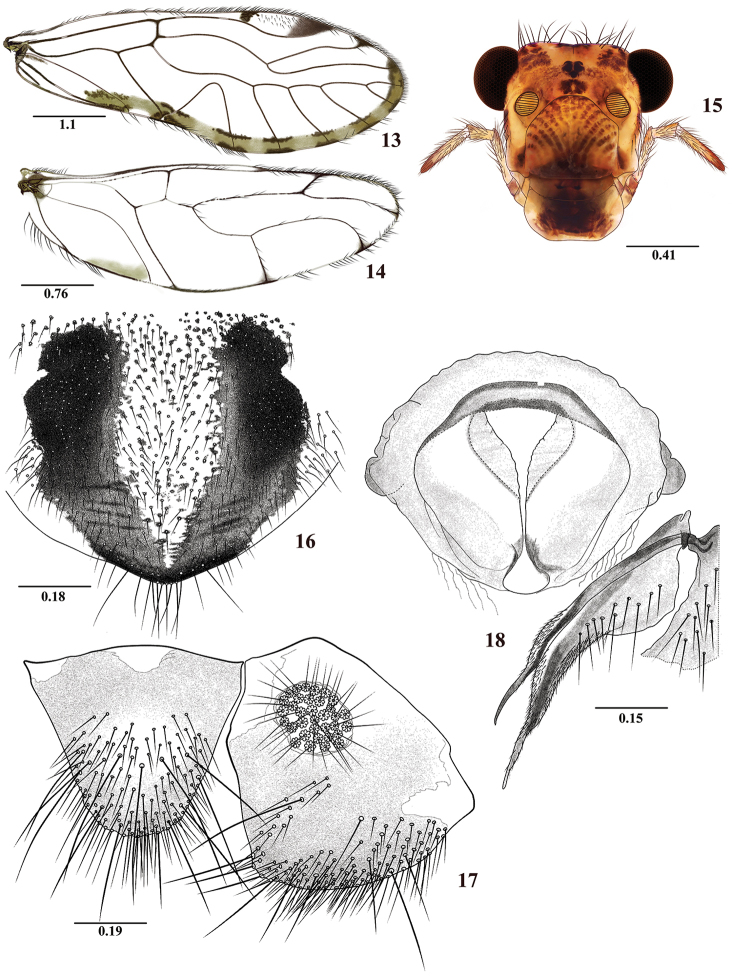
*Colocanianorsantanderina* sp. n. Female. **13** Forewing **14** Hindwing **15** Front view of head **16** Subgenital plate **17** Epiproct and right paraproct **18** Sternum IX and left gonapophyses. Scale bars in mm.

**Figures 19–24.**
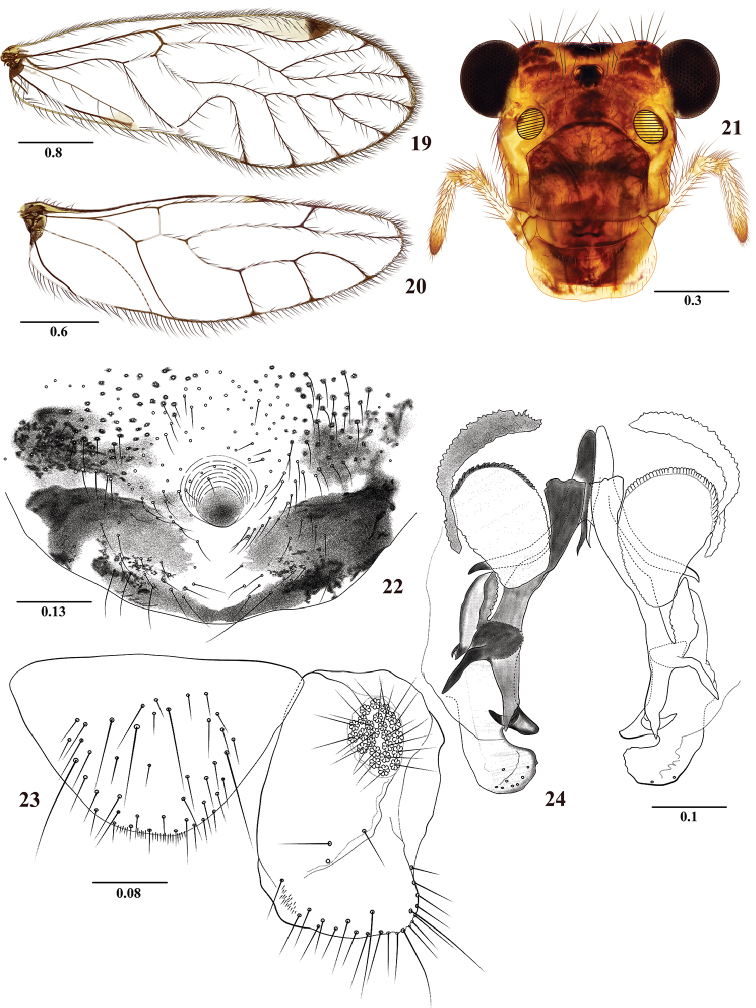
*Colocaniaoccidentalis* sp. n. Male. **19** Forewing **20** Hindwing **21** Front view of head **22** Phallosome **23** Epiproct and right paraproct **24** Hypandrium. Scale bars in mm.

**Figures 25–30.**
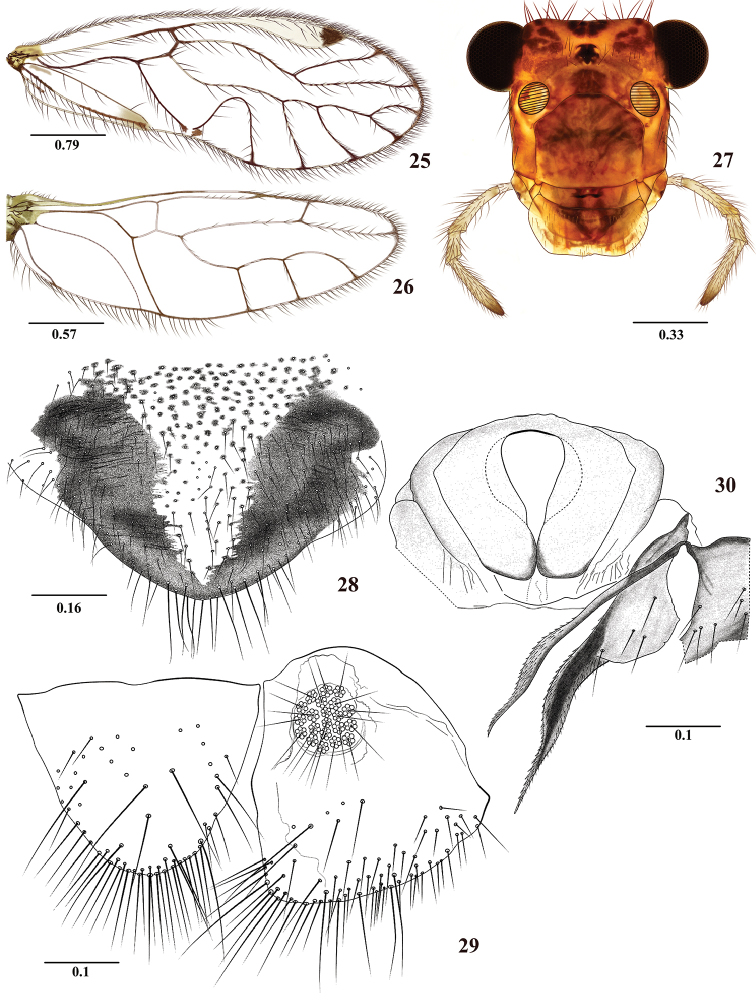
*Colocaniaoccidentalis* sp. n. Female. **25** Forewing **26** Hindwing **27** Front view of head **28** Subgenital plate **29** Epiproct and right paraproct **30** Sternum IX and left gonapophyses. Scale bars in mm.

**Figures 31–36.**
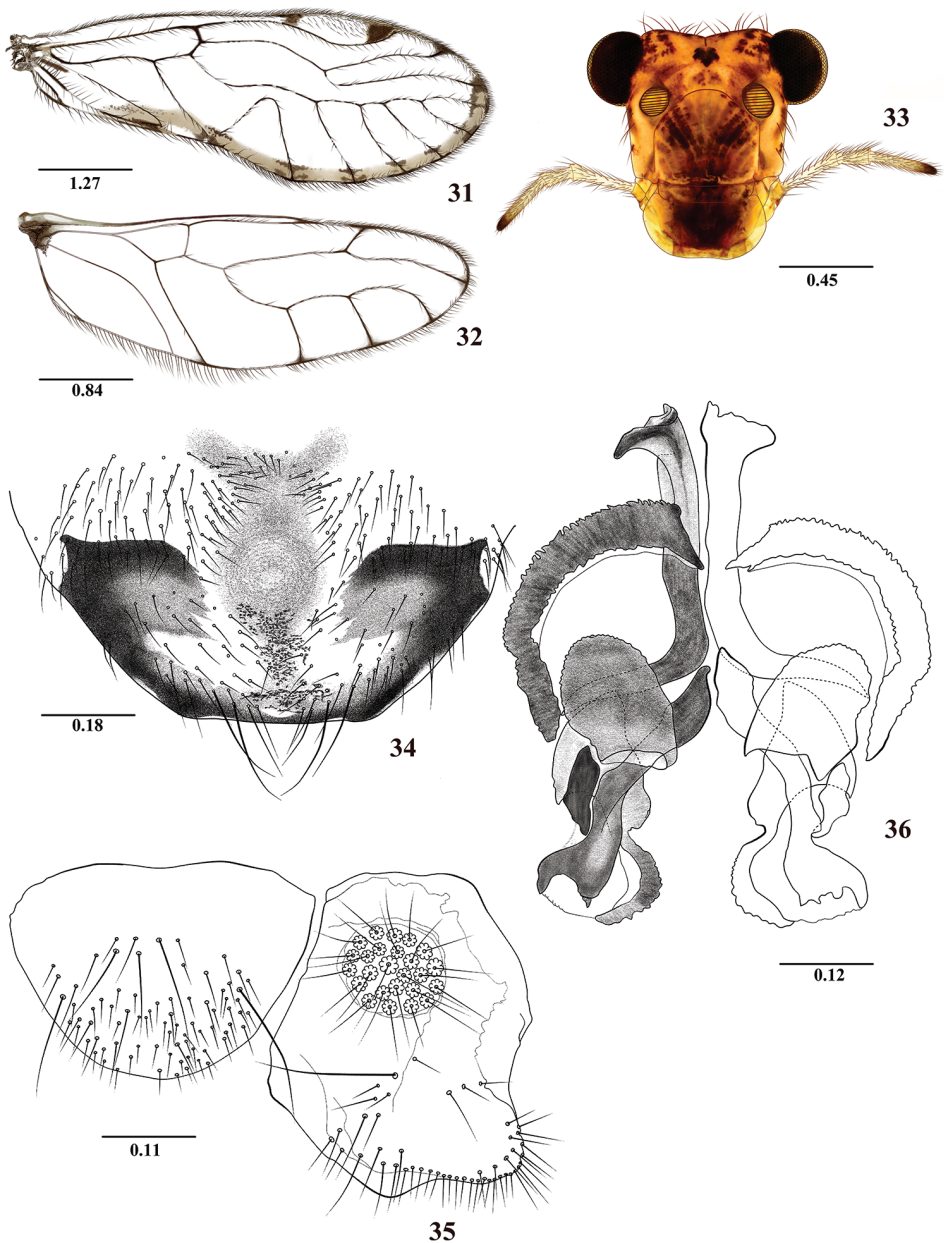
*Colocaniapilosa* sp. n. Male. **31** Forewing **32** Hindwing **33** Front view of head **34** Hypandrium **35** Left paraproct and epiproct **36** Phallosome. Scale bars in mm.

**Figures 37–42.**
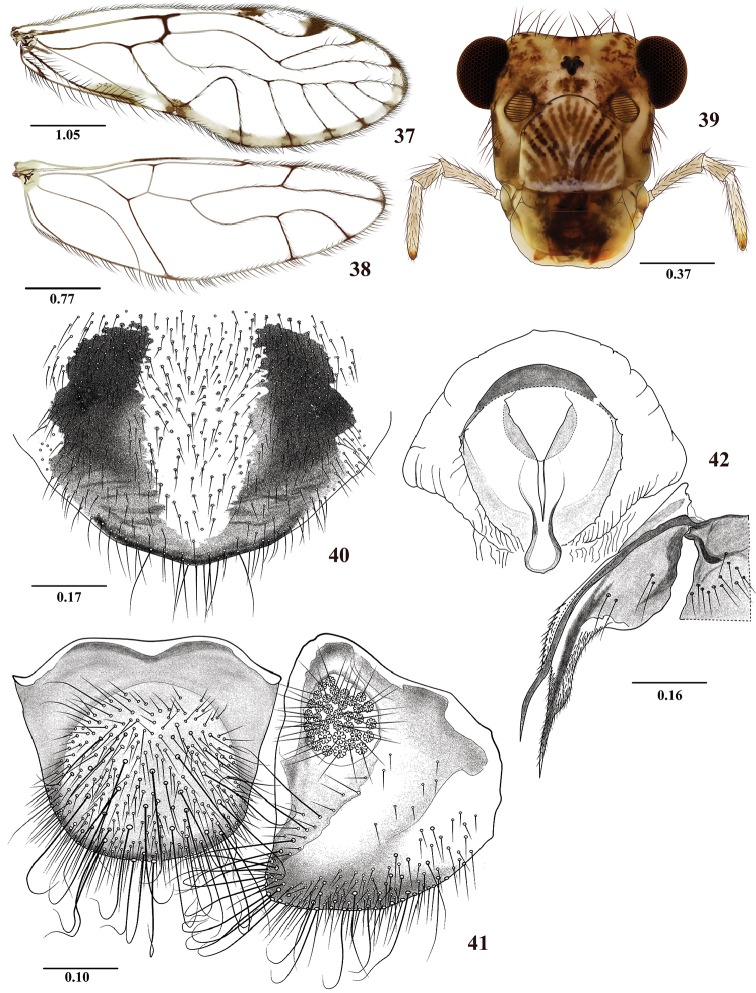
*Colocaniapilosa* sp. n. Female **37** Forewing **38** Hindwing **39** Front view of head **40** Subgenital plate **41** Epiproct and right paraproct **42** Sternum IX and left gonapophyses. Scale bars in mm.

The 19 characters used by Silva Neto et al. (2016b), plus four that are diagnostic of the species of *Colocania* gen. n., were used in the analysis, which generated a matrix with 391 cells and only three cells with inapplicable values. The additional characters are:

20. Costal margin, between base and nodus: (0) straight (1) convex (Figures 1, 7, 13, 19, 25, 31, 37).

21. Shape of proximal area of pterostigma (at around 1/3 of its length): (0) not petiolated (Figure 43), (1) little petiolated (Figure 44), (2) strongly petiolated (Figures 1, 7, 13, 19, 25, 31, 37).

22. Anterior region of hypandrium: (0) without ringed, short digitiform process, (1) with vestigial or short ringed digitiform process (Figures 4, 10, 22, 34).

23. Oval cuticular depression on central-median area of clunium: (0) absent, (1) present (Figures 45, 46).

## Results

### Colocania

Taxon classificationAnimaliaPsocodeaPtiloneuridae

González, García Aldrete & Mendivil gen. n.

http://zoobank.org/AC79B1A9-F003-4888-BB01-C9FE4D68CFE5

#### Type species.

*Colocaniaoccidentalis* González, García Aldrete & Mendivil, sp. n.

#### Etymology.

The generic name is a compound word, formed with the root “colo” from Colombia, and the suffix “cania”, common in Ptiloneuridae, as in *Euplocania*, *Perucania*, *Triplocania*. It refers to its endemicity in Colombia.

#### Diagnosis.

Forewings with costal margin, between base and nodus, strongly to gently convex, pterostigma elongate, petiolate proximally at around 1/3 of its length, Rs curved strongly towards pterostigma; anterior area of the hypandrium with a short ringed digitiform process. Phallosome most commonly with two separate stems proximally usually projected to the hypandrium (Figures 6, 24, 36). Clunium with oval cuticular depression on central-median area. Females with sternum IX oval to subtrapeziform (Figures 18, 30, 42), with a generally globose or pear-shaped area in the middle.

##### Key to the genera of Ptiloneuridae (males) modified from García Aldrete (2006), Silva-Neto and García Aldrete (2015), and Silva Neto et al. (2016a)

**Table:** 

1	Hindwing M one-branched	**2**
–	Hindwing M two to five-branched	**7**
2	Forewing 2A joining wing margin; no crossveins between 2A and wing margin	**3**
–	Forewing 2A joining 1A; one crossvein between 2A and wing margin; two crossveins between 1A and wing margin	***Timnewia* García Aldrete**
3	Forewing areola postica high, with apex rounded	**4**
–	Forewing areola postica low, very long	***Perucania* New & Thornton**
4	Labral sclerites incomplete, not reaching anterior margin of labrum	**5**
–	Labral sclerites complete, almost reaching anterior margin of labrum	***Willreevesia* García Aldrete**
5	Forewing M three-branched, occasionally M3 forked	***Triplocania* Roesler**
–	Forewing M more than three-branched	**6**
6	Forewing M four to five-branched	***Euplocania* Enderlein**
–	Forewing M six-branched	***Omilneura* García Aldrete**
7	Forewing areola postica free, high, with apex rounded	**8**
–	Forewing areola postica high, rigidly triangular, joined to M by a crossvein	***Ptiloneuropsis* Roesler**
8	Forewing 2A without one crossvein to wing margin pterostigma long, smooth	**9**
–	Forewing 2A with one crossvein to wing margin, pterostigma long, distinctly spurred	***Loneuroides* García Aldrete**
9	Forewing with costal margin, between the base and the nodus strongly to gently convex, pterostigma proximally petiolate at around 1/3 of its length, Rs strongly curved towards pterostigma; hypandrium strongly convex, of one piece; anterior area of the hypandrium with a short ringed digitiform process, sometimes visible only by the presence of concentric rings	***Colocania* gen. n.**
–	Forewing with costal margin between the base and the nodus almost straight, pterostigma proximally little petiolate; Rs slightly curved towards pterostigma or almost straight; hypandrium simple or with three or more well-defined sclerites	**10**
10	Forewing M four to seven-branched; hindwing M two to five-branched	**11**
–	Forewing M eight-branched; hindwing M five-branched; hypandrium a broad sclerite projected posteriorly to form a wide, three lobed sclerite, with a dense field of setae on each postero-lateral corner, and a dense field of setae on each side of the central projection	***Ptiloneura* Enderlein**
11	Hypandrium simple, of one sclerite; hindwing M two to four-branched; phallosome with external parameres distally forked or rounded, enclosing a membrane with numerous pores	***Brasineura* Silva Neto & García Aldrete**
–	Hypandrium simple or formed by a central sclerite and one or two smaller ones on each side; hindwing M two to five-branched; phallosome with external parameres without a membranous region with numerous pores	***Loneura* Navás**

##### Position of *Colocania* gen. n. in Ptiloneuridae

From 88 most parsimonious cladograms, obtained for each *K* value analyzed (*K* = 1–9), 17 consensus topologies were retained (L = 75–76, CI = 48–49, RI = 61–62). In all of them the clade formed by the species of *Colocania* was maintained, although between *K* = 5–9 the relations of this with the rest of the genera is unstable. This however, remains constant between *K* = 1–4 (L = 76, CI = 48, RI = 61), which includes the value calculated as appropriate (*K* = 2) through the use TNT script (setk.run) on the data set. In this way, the strict consensus topology obtained in the cladistic analyses with different optimality criteria of parsimony, showed that *Colocania* is a monophyletic group, supported by high symmetric resampling (88%) and Bootstrap (82%) (Figure 47). This monophyly is supported by two unambiguous synapomorphies: costal margin, between base and nodus, convex (char. 20:1), and clunium with oval cuticular depression on central-median area (char. 23:1), present in both males and females (Figures 45, 46). This genus is also supported by the homoplasic condition: pterostigma proximally petiolate for around 1/3 of its length (char. 21:2), external parameres stout (char. 17:0); and absence of a mesal transverse endophallic sclerite (char. 18:0). It is related to *Ptiloneuropsisdiamantina*, forming a clade supported by one unambiguous synapomorphy: anterior region of hypandrium with vestigial or short ringed digitiform process (char. 22:1) and by a homoplasic condition: marginal area of the forewing from R4+5 to Cu1a hyaline (char. 10:0). The clade above is related to a clade conformed by *Loneura*, *Loneuroides* and *Ptiloneura*, forming a clade supported by one unambiguous synapomorphy: hindwing with four primary branches in vein M, which could change in future analyzes if we consider all the possible variations that can be observed in the species of these three genera. Furthermore, this clade, as well as the related to the other genera, showed unstable arrangements when the characters tend to have the same weight (K = 5–9) (cladograms not shown). Silva Neto et al. (2016) recognized two distinct clades that group the genera of Ptiloneuridae; one of them includes *Belicania*, *Euplocania*, *Omilneura*, *Perucania*, *Timnewia*, and *Triplocania*. The other clade includes *Brasineura, Loneura, Loneuroides, Ptiloneura, Ptiloneuropsis*, and *Willreevesia*. In the latter, *Brasineura* and *Loneuroides* are recognized as monophyletic, while in the first, the sub-clade formed by *Omilneura*, *Perucania*, *Timnewia*, and *Triplocania*, that according to our results and given the high frequency of homoplasic characters seem to require additional phylogenetic analysis, within the family Ptiloneuridae, including in each genus a greater number of species and evaluating a greater number of characters.

### Colocania candelaria

Taxon classificationAnimaliaPsocodeaPtiloneuridae

García Aldrete, González & Carrejo sp. n.

http://zoobank.org/D0D430EE-7F4A-4284-9E61-261830D9559E

Figures 1–6

#### Type locality.

**COLOMBIA.** Huila. Belén, La Candelaria, 2128 m. 02°13'36.0"N, 76°07'27.4"W.

#### Type material.

Holotype male. 18.IV.2015. On tree trunk. R. González. Deposited in Entomological Museum, Universidad del Valle (MUSENUV, slide code 29033).

#### Diagnosis.

Forewings hyaline, without marginal bands as in *C.chicaque* sp. n., and *C.occidentalis* sp. n., differing from them by wing characters, phallosome, and details of hypandrium and epiproct. Unlike the above species, the pterostigma has large pigmented bands proximally and distally (Figure 1); phallosome with mesal endophallic sclerite widened, apically narrow and with two elongated teeth, one of them curved outwards (Figure 6).

#### Description.

**Color** (in 80% ethanol). Body pale brown, with pigmented dark brown areas as indicated below. Compound eyes black, ocelli hyaline, with ochre centripetal crescents. Vertex with three dark brown areas, a central one and two lateral ones between the compound eyes. Front with brown area between ocellar group and epistomal sulcus, as illustrated (Figure 3). Postclypeus brown, with diagonal dark brown stripes. Genae, anteclypeus, labrum, mandibles, maxillae, and labium brown to pale brown. Antennae brown, scape pale brown, flagellomeres distally cream. Maxillary palps pale brown, Mx4 dark brown distally. Tergal lobes of meso- and metathorax brown. Thoracic pleura pale brown. Mesopleura with dark brown spots. Legs: coxae, trochanter, and femora pale brown, tibiae and tarsi brown. Wings hyaline, forewing pterostigma with a large dark brown band proximally and distally. Abdomen cream, with subcuticular brown spots; clunium, hypandrium, and phallosome dark brown; epiproct and paraprocts brown.

#### Morphology.

As in diagnosis, plus the following: Head elongate: H/MxW: 1.52; small compound eyes, H/d: 4.24; H/D: 3.1, IO/MxW: 0.80. Upper ends of compound eyes almost reaching the level of the vertex. Outer cusp of lacinial tip broad, with six denticles. Mx4/Mx2: 1.13. Forewing (Figure 1): L/W: 2.72. Pterostigma: lp/wp: 5.97, areola postica tall, triangular; al/ah: 1.27, R_4+5_ almost straight, M five-branched, M_5_ distally forked. Hindwings (Figure 2): l/w: 3.08. M four branched. Hypandrium (Figure 4), with three pigmented areas, two antero-lateral, curved, backwards and a central, posterior one, wide and narrow, setose as illustrated; phallosome with side struts independent, with two separate basal stems anteriorly wide, narrowing distally and curved outwards, basally articulated to a mesal process that projects to the hypandrium; external parameres laminar, dilated distally, apex rounded, bearing pores (Figure 6); anterior pair of endophallic sclerites oval, antero-lateral pair curved as illustrated, rounded distally and overlapping with the basal part of the external parameres (Figure 6). Paraprocts (Figure 5) broad, elliptic, with distal setal field as illustrated, sensory fields oval, with 34 trichobothria on basal rosettes. Epiproct (Figure 5) broadly trapeziform, with a group of three mesal macrosetae and a setal field distally on each side; posterior border with a field of microsetae and a row of four-five setae.

#### Measurements.

FW: 6250, HW: 4075, F: 1470, T: 2560, t1: 900, Mx4: 360, ctt1: 26, f1: 1230, f2: 1240, f3: 1000, f4: 760, f5: 480, f6: 410, f7: 320, f8: 295, f9: 210, f10: 200, IO: 580, D: 360, d: 260, IO/d: 2.23, PO: 0.72.

#### Etymology.

The specific epithet refers to the town of La Candelaria (La Plata, Huila) where the holotype was collected.

### Colocania chicaque

Taxon classificationAnimaliaPsocodeaPtiloneuridae

González, García Aldrete & Carrejo sp. n.

http://zoobank.org/3A56FDF4-B85B-4F86-88E8-0F535E8A5B5A

Figures 7–12

#### Type locality.

COLOMBIA. Cundinamarca. Soacha, Chicaque Natural Reserve, Tirolesa, 2240 m. 04°36'40.38"N, 74°18'41.7"W.

#### Type material.

Holotype male. 24–28.II.2014. Malaise trap. D. Forero. Deposited at the Pontífica Universidad Javeriana Museum (MPUJ). Bogotá, Colombia.

#### Diagnosis.

Forewings hyaline, without marginal bands as in *C.candelaria* sp. n., and *C.occidentalis* sp. n., differing from them by characters of the forewing, phallosome and details of hypandrium and epiproct. Unlike *C.candelaria* the pterostigma has only a distal pigmented band, sometimes with a median, little defined, small pigmented area (Figure 7); phallosome with narrow mesal endophallic sclerite, with distal processes curved as illustrated. Unlike *C.occidentalis*, the forewing has M five-branched, with M5 distally forked (Figure 7).

#### Color

(in 80% ethanol). Body pale brown, with pigmented dark brown areas as indicated below. Compound eyes black, ocelli hyaline, with ochre centripetal crescents. Vertex with brown areas, central and lateral, between compound eyes. Front brown as illustrated (Figure 9). Postclypeus brown, with diagonal dark brown bands. Genae, anteclypeus, labrum, mandibles, maxillae and labium pale brown. Maxillary palps pale brown, Mx4 distally dark brown. Tergal lobes of meso- and metathorax brown. Thoracic pleura creamy to pale brown. Legs pale brown. Wings hyaline, forewing pterostigma as in diagnosis, veins brown. Abdomen pale brown, with subcuticular spots brown; clunium brown, clearer dorsally, with tubercle brown, hypandrium, phallosome, epiproct, and paraprocts brown.

#### Morphology.

As in diagnosis, plus the following: Head elongate: H/MxW: 1.48, H/D: 3.0; H/d: 4.0; IO/MxW: 0.74. Vertex slightly emarginated; upper ends of compound eyes almost reaching the level of the vertex. Outer cusp of lacinial tip broad, with eight denticles. Mx4/Mx2: 1.09. Forewing (Figure 7): L/W: 2.73. Pterostigma elongate: lp/wp: 5.88, areola postica tall, triangular: al/ah: 1.24, asymmetric; hindwings (Figure 8): l/w: 3.10, M four-branched. Hypandrium (Figure 10), setose as illustrated. Phallosome with side struts V-shaped, distally with arms curved outward; external parameres laminar, dilated distally, apex rounded, bearing pores (Figure 12); anterior pair of endophallic sclerites laminar-oval, with anterior margin serrate; antero-lateral pairs boomerang-shaped, curved inward proximally and with a series of laminar teeth, distally without teeth and reaching near the basal part of the external parameres (Figure 12). Paraprocts (Figure 11) robust, almost elliptic, with distal setal field as illustrated, sensory fields oval, with 26 trichobothria on basal rosettes. Epiproct (Figure 11) trapeziform, with a group of three mesal macrosetae and a setal field distally.

#### Measurements.

FW: 6700, HW: 4500, F: 1610, T: 2760, t1: 1240, t2: 100, t3: 152, Mx4: 380, ctt1: 42, f1: 1480, f2: 1380, IO: 600, D: 400, d: 300, IO/d: 2, PO: 0.75.

#### Etymology.

The specific epithet refers to the Chicaque Natural Reserve, where the holotype was collected.

### Colocania norsantanderina

Taxon classificationAnimaliaPsocodeaPtiloneuridae

Carrejo, Mendivil & González sp. n.

http://zoobank.org/DDB056B0-536F-43B6-AEAD-3E9D62DE29E5

Figures 13–18

#### Type locality.

COLOMBIA. Norte de Santander. National Natural Park Tamá. Orocue Station, 2433 m. 07°25'27.6"N, 72°26'31.8"W.

#### Type material.

Holotype female. 2.VII.2016. On wood beam with mosses and fungi. R. González, N. Carrejo. Deposited in Entomological Museum, Universidad del Valle (MUSENUV, slide code 29034), Santiago de Cali, Colombia.

#### Diagnosis.

Related to *C.pilosa* sp. n. in having the forewing with similarly pigmented pattern and shape of the areola postica. It differs from the female of *C.pilosa* by having the hindwing M widely forked, by the shape of sternum IX, and by the shape and pilosity of the epiproct, without distally curved setae.

#### Color

(in 80% ethanol). Body pale brown, with pigmented dark brown areas as indicated below. Compound eyes black, ocelli hyaline, with ochre centripetal crescents. Vertex and front with brown spots, central and lateral between the compound eyes (Figure 15). Postclypeus pale brown, with diagonal bands dark brown. Genae, anteclypeus, mandibles, maxillae, and labium pale brown, labrum brown. Antennae brown, scape and pedicel pale brown, flagellomeres cream apically. Maxillary palps pale brown, Mx4 brown distally. Tergal lobes of meso- and metathorax brown. Thoracic pleura creamy to pale brown with brown spots. Legs: coxae dark brown, fore- coxae pale brown, trochanters cream to pale brown, femora pale brown, with brown spots, tarsi 1 brown apically, tarsi 2–3 brown. Forewing with marginal brown band from apex of R4+5 to A1; M five branched; pterostigma with a dark brown band proximally and distally. Abdomen pale brown, with subcuticular brown spots; clunium brown, paler dorsally, with tubercle brown, subgenital plate with V-shaped pigmented area (Figure 16); gonapophyses, paraprocts, and epiproct dark brown.

#### Morphology.

As in diagnosis, plus the following: Head elongate (Figure 15): H/MxW: 1.41; H/D: 3.0, H/d: 4.0: IO/MxW: 0.78. Outer cusp of lacinial tip broad, with seven denticles. Mx4/Mx2: 1.14. Forewing (Fig. 13), L/W: 2.83. Pterostigma elongate: lp/wp: 5.73, areola postica triangular, al/ah: 1.35, M five branched. Hindwings (Figure 14): l/w: 2.89, M two branched. Subgenital plate (Figure 16) broad, slightly pointed posteriorly, setose, with macrosetae posteriorly. Gonapophyses (Figure 18): v1 elongate, pilose, distally acuminate; v2+3 pilose distally, with a row of 10 setae on v3; distal process acuminate, with microsetae on surface. Paraprocts (Figure 17) triangular, rounded apically, distal setal field with abundant setae as illustrated, sensory fields with 35 trichobothria on basal rosettes. Epiproct (Figure 17) triangular, rounded apically, with abundant setae and macrosetae distally as illustrated.

#### Measurements.

FW: 6075, HW: 3975, F: 1460, T: 2700, t1: 1170, t2: 105, t3: 160, Mx4: 375, ctt1: 46, f1: 1320, f2: 1330, f3: 1030, IO: 620, D: 370, d: 273, IO/d: 2.27, PO: 0.74.

#### Etymology.

The specific epithet refers to the Colombian Department Norte de Santander, where the holotype was collected.

### Colocania occidentalis

Taxon classificationAnimaliaPsocodeaPtiloneuridae

González, García Aldrete & Mendivil sp. n.

http://zoobank.org/3991BED3-1F9B-4406-9035-19D8CBF63D6D

Figures 19–24 , 25–30

#### Type locality.

COLOMBIA. Valle del Cauca. Santiago de Cali, El Saladito, San Antonio, 2142m. 03°29'23.5"N, 76°37'39.4"W.

#### Type material.

Holotype male. 27.I.2012. On tree trunks covered with lichens and mosses. R. González. Deposited in Entomological Museum, Universidad del Valle (MUSENUV, slide code 29035), Santiago de Cali, Colombia. Paratypes: 2 males, same data as the holotype, J. Mendivil & R. González. 1 male, 2 females, Valle del Cauca, Santiago de Cali, Los Andes-Charco Azul, 1687 m. 03°25'21.7"N, 76°37'0.1"W, 23.I.2013. R. González (Female: MUSENUV, slide code 29036). 2 males, 2 females, Los Andes-Quebrada Honda, 1900 m. 03°26'01.8"N, 76°38'40.3"W, 23.I.2013. R. González. 1 male, 1 female, Risaralda, Santuario, Planes de San Rafael, 2092 m. 05°07'13.9"N, 76°00'04.5"W, R. González (male: MUSENUV, slide code 29037). All paratypes on tree trunks covered with lichens and mosses.

#### Diagnosis.

Forewings hyaline, without marginal pigmented band as in *C.candelaria* sp. n., and *C.chicaque* sp. n., differing from them by having the forewing M four-branched (Figure 19), and the hindwing M three-branched (Figure 20), and by details of the phallosome and hypandrium.

#### Male.

**Color** (in 80% ethanol). Body cream to pale brown, with pigmented dark brown areas as indicated below. Compound eyes black, ocelli hyaline, with ochre centripetal crescents. Vertex with dark brown spots, central and lateral between compound eyes. Front with brown spots between ocellar group and epistomal sulcus, as illustrated (Figure 21), with pale cream band parallel to the antennal sockets. Postclypeus brown, with diagonal pale brown bands. Anteclypeus and labrum pale brown. Genae brown. Postgena pale cream. Antennae brown, flagellomeres cream apically. Maxillary palps pale brown, Mx4 dark brown distally. Tergal lobes of meso- and metathorax dark brown; thoracic pleura dark brown with pale spots. Legs pale brown, coxae with brown spots basally, femora with gray-brown ring basally and gray-brown spots widely distributed; tibiae with gray-brown spot, tarsi 1 brown apically; tarsi 2–3 brown. Wings hyaline, forewing pterostigma with one distal large dark brown band, some with additional proximal spot (not illustrated). Abdomen pale cream, with subcuticular bands brown; clunium and hypandrium brown; clunium with pale central area, hypandrium anteriorly cream; phallosome brown, with sclerites dark brown; epiproct and paraprocts brown.

#### Morphology.

As in diagnosis, plus the following: Head elongate (Figure 21): H/MxW: 1.44; H/D: 3.1, H/d: 4.19; IO/MxW: 0.78. Outer cusp of lacinial tip broad, with nine denticles. Mx4/Mx2: 1.10. Forewings (Figure 19): L/W: 2.69. Pterostigma elongate: lp/wp: 6.91, areola postica tall, triangular, with apex rounded: al/ah: 1.52; M four-branched. Hindwings (Figure 20): l/w: 3.02; M three branched. Hypandrium with three pigmented, setose areas, the two anterior widely separated and weakly connected to the posterior area, the latter narrow and extended laterally as a boomerang of little curvature (Figure 22). Phallosome (Figure 24) with laminar external parameres, wide, with apically rounded lobe bearing pores, curved inwards; mesal sclerite process with curved outwards apical teeth as illustrated. Side struts V-shaped, basally with an attached complex structure that projects over the hypandrium, distally curved outwards. Anterior endophallic sclerites laminar and oval, with serrate anterior margin, lateral sclerites curved as illustrated. Paraprocts (Figure 23) almost elliptic, with a distal setal field; sensory fields with 24 trichobothria on basal rosettes. Epiproct (Figure 23) semi-oval, rounded posteriorly, setal field with setae and macrosetae, posterior margin with small setae as illustrated.

#### Measurements.

FW: 4375, HW: 2987, F: 1310, T: 2050, t1: 990, t2: 96, t3: 135, Mx4: 320, ctt1: 38, f1: 1000, f2: 990, f3: 770, f4: 510, f5: 355, f6: 300, f7: 240, f8: 200, f9: 176, f10: 150, f11: 180, IO: 535, D: 320, d: 235, IO/d: 2.28, PO: 0.73.

#### Female.

**Color** (in 80% ethanol). Body, head, legs, epiproct, paraprocts, and wings as in the male, plus the following: Subgenital plate with pigmented area V-shaped, arms wider proximally. Clunium, epiproct and paraprots brown. Gonapophyses dark brown. Sternum IX cream yellowish, darker on the edges.

#### Morphology.

As in diagnosis, plus the following: Head elongate (Figure 27): H/MxW: 1.46; H/D: 3.3, H/d: 4.43; IO/MxW: 0.81. Outer cusp of lacinial tip broad, with eight denticles. Mx4/Mx2: 1.14. Wings (Figures 25 and 26) as in the male, L/W: 2.72. Pterostigma: lp/wp: 5.08, areola postica: al/ah: 1.23. Hindwings (Figure 26): l/w: 3.00. Subgenital plate (Figure 28) broad, posteriorly rounded, setose, with apical macrosetae as illustrated. Gonapophyses (Figure 30): v1 elongate, acuminate, distally with microsetae; v2+3 with short anterior heel, with four setae on v3; distal process sinuous, acuminate, bearing microsetae. Sternum IX broad, convex anteriorly, with a median concavity posteriorly, and a central, rounded mesal area as illustrated (Figure 30). Paraprocts (Figure 29) triangular, distal setal field with abundant setae and macrosetae as illustrated, sensory fields with 22–24 trichobothria on basal rosettes. Epiproct (Figure 29) triangular, with rounded apex, with abundant macrosetae distally, particularly on posterior margin as illustrated.

#### Measurements.

FW: 4350, HW: 2925, F: 1300, T: 2050, t1: 940, t2: 104, t3: 124, Mx4: 330, ctt1: 35, f1: 1130, f2: 1100, f3: 820, f4: 580, f5: 365, f6: 290, f7: 210, IO: 570, D: 312, d: 230, IO/d: 2.48, PO: 0.74.

#### Etymology.

The specific name refers to the distribution of the species in localities of the western Andean Cordillera in Colombia.

### Colocania pilosa

Taxon classificationAnimaliaPsocodeaPtiloneuridae

González, Carrejo & García Aldrete sp. n.

http://zoobank.org/5440EA82-CAED-4844-B047-82C2EC811212

Figures 31–36 , 37–42

#### Type locality.

COLOMBIA. Huila. La Plata, Belén, Meremberg Nature Reserve, 2352 m. 02°13'06.6"N, 76°07'01.1"W.

#### Type material.

Holotype male. 20.I.2015. On tree trunks covered with lichens and mosses. R. González. Deposited in Entomological Museum, Universidad del Valle (MUSENUV, slide code 29038), Santiago de Cali, Colombia. Paratype female, 28.X.2016. Same data as the holotype (MUSENUV, slide code 29039).

#### Diagnosis.

It is related to *C.norsantanderina* sp. n., described above (see diagnosis of the latter). Both species seem to have a sister-group relationship, to be confirmed when the male of the latter be found. The ninth sterna and gonapophyses in both species are quite similar, but the epiprocts are distinct. The male presents side struts with arms independent, proximally paralell, with processes directed outwards, unique among the species of the genus, distally curved outwards and not fused to the external parameres (Figure 36).

#### Male.

**Color** (in 80% ethanol). Head pattern (Figure 33). Compound eyes black, ocelli hyaline, with ochre centripetal crescents. Front and postclypeus pale brown, anteclypeus and labrum dark brown centrally, with sides pale brown. Genae pale brown with brown spots. Antennae brown, scape, pedicel and flagellomeres 1–3 pale brown, cream distally. Maxillary palps creamy, Mx4 distally brown. Tergal lobes of meso- and metathorax pale brown, with brown spots. Thoracic pleura creamy to pale brown, with brown spots. Legs pale brown, tarsi 2–3 dark brown, fore- coxae cream, middle- and hind- coxae brown, darker proximally, femora with brown spots. Forewing with marginal brown band from apex of R4+5 to A1; M five-branched; pterostigma with a proximal and a distal brown band. Hindwings hyaline, veins brown. Abdomen pale brown to cream, with subcuticular brown spots; clunium, epiproct, and paraprocts brown, epiproct with large, pale central area. Sclerites of hypandrium and phallosome dark brown.

#### Morphology.

As in diagnosis, plus the following: Head elongate (Figure 33): H/MxW: 1.37; compound eyes small, H/d: 3.94, H/D: 2.9, IO/MxW: 0.78. Outer cusp of lacinial tip broad, with eight denticles. Mx4/Mx2: 0.98. Forewings (Figure 31): L/W: 2.85. Pterostigma elongate: lp/wp: 5.56, areola postica tall, triangular, with rounded apex: al/ah: 1.40. M five-branched. Hindwings (Figure 32): l/w: 2.88. M three-branched. Hypandrium broadly trapeziform, setose, pigmented as illustrated in Figure 34. Phallosome (Figure 36), mesal endophallic sclerites elongate, shaped as an inverted V, basally separated, wide apically (mesal sclerite process) and overlapping on the external parameres and with short processes of rounded apices, with ante-apically lateral mesal sclerite process as illustrated; anterior endophallic sclerites oval and laminar, antero-lateral sclerites curved, boomerang-shaped as illustrated. Paraprocts (Figure 35) almost elliptic, with abundant setal field distally; sensory fields with 30 trichobothria on basal rosettes. Epiproct (Figure 35) semioval, rounded posteriorly, bearing macrosetae; lateral and mesal macrosetae as illustrated.

#### Measurements.

FW: 6475, HW: 4025, F: 1450, T: 2570, t1: 1180, t2: 120, t3: 160, Mx4: 325, ctt1: 36, f1: 1150, f2: 1120, f3: 900, f4: 690, f5: 410, f6: 360, f7: 260, f8: 230, f9: 200, f10: 173, f11: 200, IO: 610, D: 370, d: 270, IO/d: 2.26, PO: 0.73.

#### Female.

**Color** (in 80% ethanol). Body, head, legs, wings, epiproct and paraprocts as in the male, plus the following: pigmented area of subgenital plate U-shaped (Figure 40); gonapophyses and paraprocts dark brown, epiproct brown.

#### Morphology.

As in diagnosis, plus the following: Head elongate (Figure 39): H/MxW: 1.43; H/D: 3.2, H/d: 4.48; IO/MxW: 0.79. Outer cusp of lacinial tip broad, with seven denticles. Mx4/Mx2: 1.15. Wings (Figures 37 and 38) as in the male, L/W: 2.78. Pterostigma: lp/wp: 5.13, areola postica: al/ah: 1.31. Hindwings (Figure 38): l/w: 3.02. Subgenital plate (Figure 40) broad, slightly pointed posteriorly, setose. Gonapophyses (Figure 42): v1 elongate, broad, acuminate, distally bearing microsetae; v2+3 pilose, with short proximal heel, 4 setae on v3; distal process sinuous, acuminate, with microsetae on surface distally. Paraprocts (Figure 41), sensory fields with 34 trichobothria on basal rosettes. Epiproct (Figure 41) broad, scutiform, widely rounded distally, setal field with abundant setae as illustrated, macrosetae and abundant setae distally curved, as illustrated.

#### Measurements.

FW: 5550, HW: 3775, F: 1440, T: 2530, t1: 1110, t2: 114, t3: 140, Mx4: 380, ctt1: 40, f1: 1090, f2: 1115, f3: 880, IO: 620, D: 350, d: 250, IO/d: 2.48, PO: 0.71.

#### Etymology.

The specific epithet refers to the densely pilose female epiproct.

##### Key to the species of *Colocania*

**Table:** 

1	Females	**2**
–	Males	**4**
2	Forewing with a marginal pigmented band brown to yellowish from R4+5 to A1, M five branched (Figures 13, 37)	**3**
–	Forewing hyaline, without marginal pigmented band; with apical dark spot in pterostigma (Figure 25); M four branched; sternum IX as in Figure 30	***C.occidentalis* sp. n.**
3	Epiproct and paraprocts strongly setose, macrosetae strongly curved distally (Figure 41); sternum IX as in Figure 42	***C.pilosa* sp. n.**
–	Epiproct and paraprocts not as above, macrosetae not strongly curved distally (Figure 17); sternum IX as in Figure 18	***C.norsantanderina* sp. n.**
4	Forewing with a marginal pigmented band, brown to yellowish from R4+5 to A1, M five branched (Figure 31); hindwing M three branched (Figure 32); side struts of phallosome with arms independent, proximally parallel, basally with processes directed outwards (Figure 36)	***C.pilosa* sp. n.**
–	Forewing hyaline, without marginal pigmented band as above, pterostigma with proximal and distal, or only distal pigmented bands, M four-five branched (Figures 1, 7, 19); hindwing M three-four branched (Figures 2, 8, 20); side struts with arms variable	**5**
5	Forewing M four branched (Figure 19), hindwing M three branched (Figure 20); side struts of phallosome V-shaped, proximally with two separate basal elongated arms curved and projects over the hypandrium, with an attached complex structure basally (Figure 24)	***C.occidentalis* sp. n.**
–	Forewing M five branched, M5 bifurcate (Figures 1, 7); hindwing M four branched (Figures 2, 8); side struts with or without basal elongated arms that projects over the hypandrium (Figures 6, 12)	**6**
6	Pterostigma with large proximal and distal pigmented bands (Figure 1); phallosome with mesal endophallic sclerite widened, mesal sclerite processes with two elongated teeth, one of them curved outwards, side struts with two elongated separate basal arms (Figure 6)	***C.candelaria* sp. n.**
–	Pterostigma with only distal pigmented band (Figure 7); phallosome with narrow mesal endophallic sclerite, mesal sclerite processes apically toothless and curved strongly outwards, side struts V-shaped, fused basally (Figure 12)	***C.chicaque* sp. n.**

##### Comments on the species of *Colocania* gen. n.

*Colocania* is, so far, endemic to Colombia, its species have been collected between 1687 and 2450 meters of altitude, in Andean and Subandean areas (see Rangel and Aguilar 1995) of Central and East-western mountain ranges (Figure 48). The genus might also be found in Venezuela, as *C.norsantanderina* was found in a montane area near the Merida mountain range in Venezuela.

On basis of the forewing pigmentation pattern, two species groups are recognized, group A, with the forewings having a marginal pigmented band, from R4+5 to distal end of Cu2, including *C.norsantanderina* sp. n. and *C.pilosa* sp. n., and group B, with forewings hyaline, including *C.candelaria* sp. n., *C.chicaque* sp. n. and *C.occidentalis* sp. n. However, *C.candelaria* and *C.chicaque* are related only on the basis of one homoplasic character, the forewings with a ratio of crossvein Rs-M and the portion of vein M before it of 1:2 (char. 12) (Figure 47).

**Figures 43–46.**
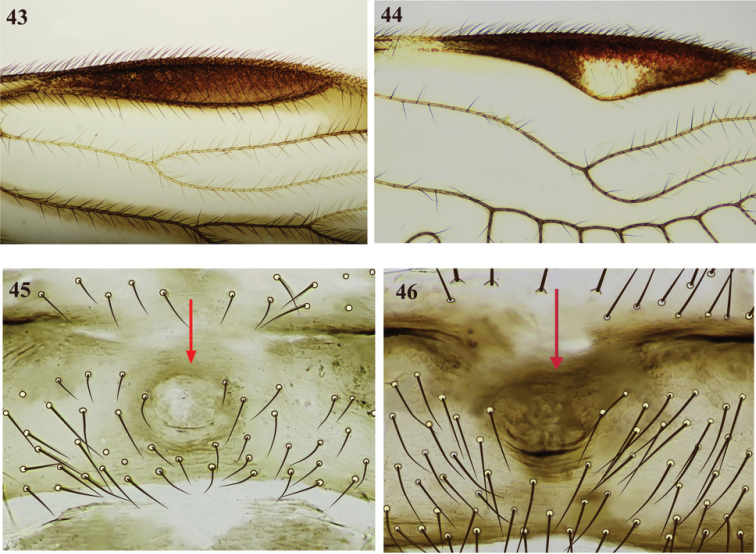
Pterostigma (**43, 44**) and central area of clunium showing oval cuticular depression (**45, 46**) **43***Perucanialongiareola***44***Ptiloneurabidorsalis***45***Colocaniacandelaria* (male) **46***C.norsantanderina* (female).

**Figure 47.**
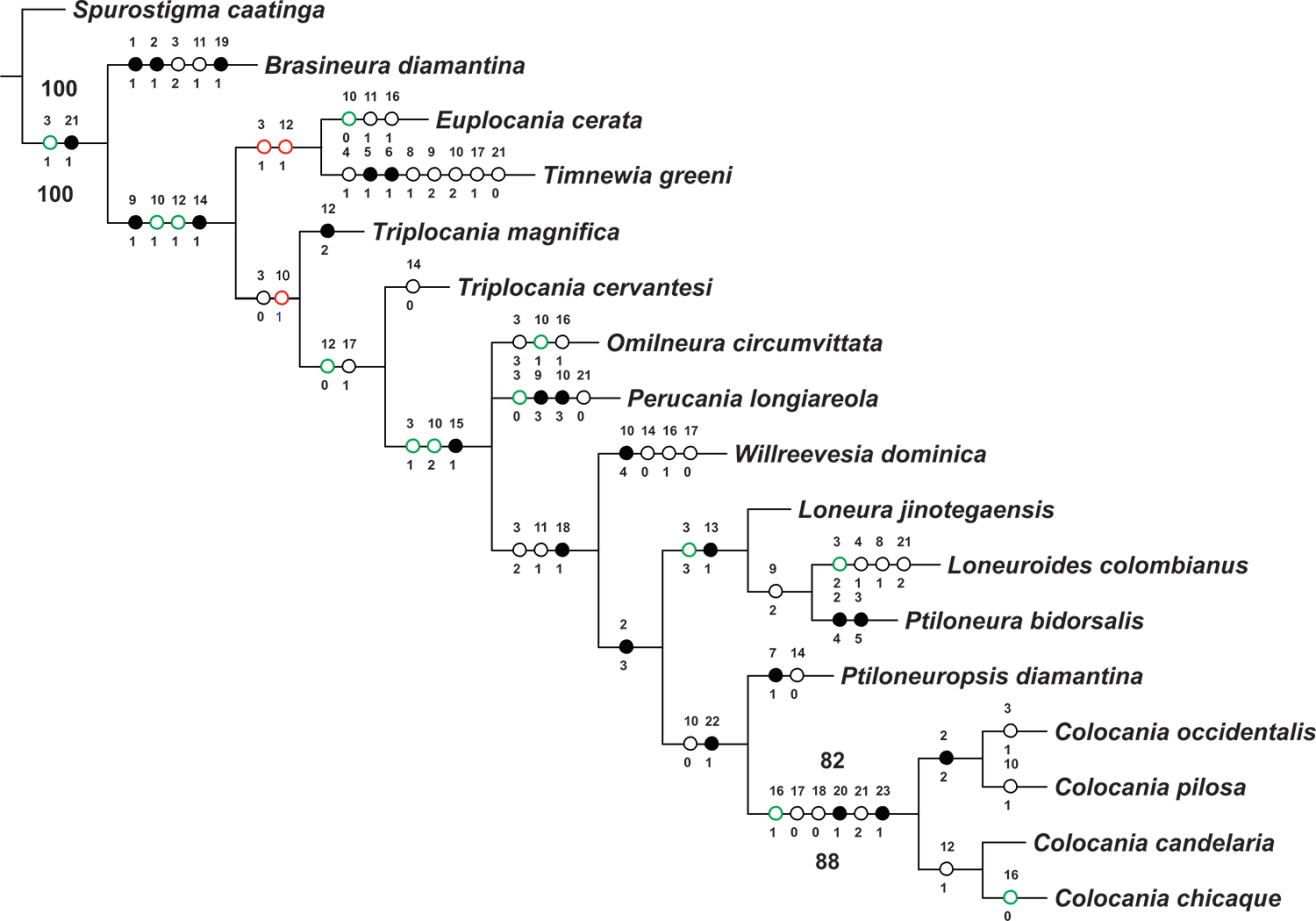
Strict consensus cladogram for 13 genera of Ptiloneuridae under implied weights for K = 2 (L = 76; IC = 48; IR = 61). Full circles (black, red, or green) indicate unique character changes, black circles (unambiguous), color circles (ambiguous); empty circles indicate parallelisms or reversals. ACCTRAN (green circles) and DELTRAN (red circles) optimization. Character number above and character states below each circle. Bootstrap (bold numbers on the branches) and Symmetric resampling (bold numbers under the branches) values>50.

**Figure 48.**
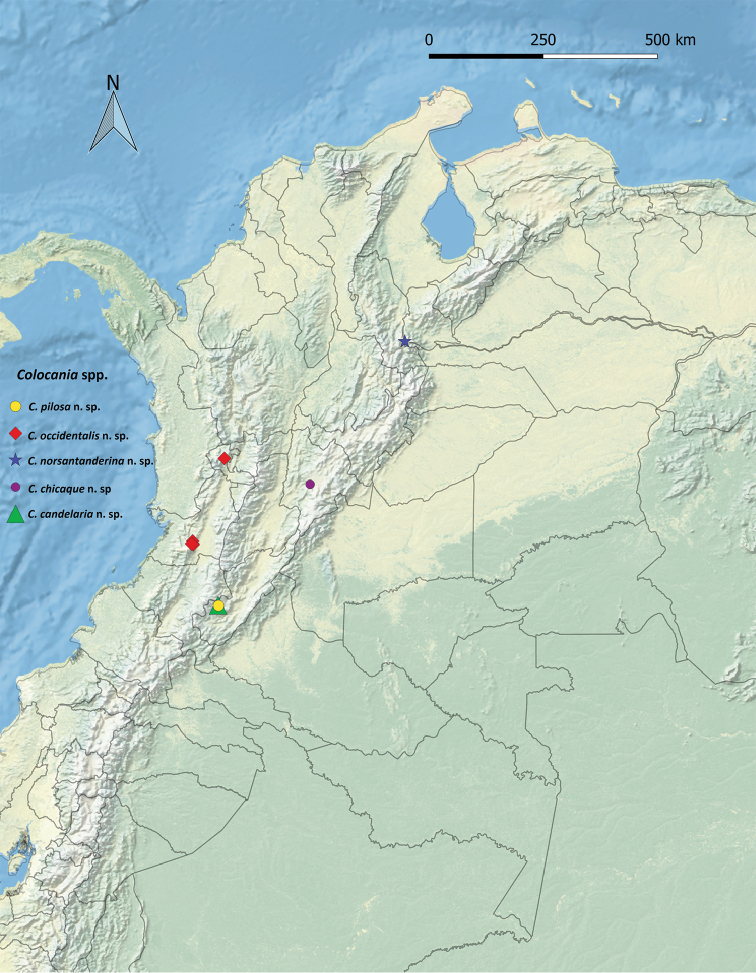
Distribution of the species of *Colocania*.

## Supplementary Material

XML Treatment for Colocania

XML Treatment for Colocania candelaria

XML Treatment for Colocania chicaque

XML Treatment for Colocania norsantanderina

XML Treatment for Colocania occidentalis

XML Treatment for Colocania pilosa
